# Recyclable Materials for Ecofriendly Technology

**DOI:** 10.3390/ma15207133

**Published:** 2022-10-13

**Authors:** Roman Fediuk, Mujahid Ali

**Affiliations:** 1 Polytechnic Institute, Far Eastern Federal University, 690922 Vladivostok, Russia; 2 Peter the Great St. Petersburg Polytechnic University, 195251 St. Petersburg, Russia; 3 Civil and Environmental Engineering Department, Universiti Teknologi PETRONAS, Seri Iskandar 32610, Perak, Malaysia; 4 Department of Civil Engineering, Faculty of Engineering, Universiti Malaya, Kuala Lumpur 50603, Malaysia

**Keywords:** Special Issue, waste recycle, building materials, ecofriendly, technology

## Abstract

This Special Issue (SI), “Recyclable Materials for Ecofriendly Technology”, has been proposed and organized as a means to present recent developments in the field of environmentally friendly designed construction and building materials. For this purpose, dozens of articles were included or considered for inclusion in this SI, covering various aspects of the topic. A comparison of these articles with other modern articles on this topic is carried out, which proves the prospects and relevance of this SI. Furthermore, per the editorial board’s journal suggestion, the second volume of this successful SI is being organized, in which authors from various countries and organizations are invited to publish their new and unpublished research work.

This Special Issue (SI), “Recyclable Materials for Ecofriendly Technology (RMET)”, was developed and organized to present new developments in the field of environmentally friendly built construction and building materials. For this reason, several articles addressing various aspects of the topic under examination were included in the Special Issue. The largest contributors to the SI were the universities and institutions considered the most successful organizations. For instance, Far East Federal University, Peter the Great St. Petersburg Polytechnic University, Belgorod State Technological University n.a. V.G. Shukhov, Universiti Teknologi PETRONAS Malaysia, COMSAT University Abbottabad, Pakistan, and M. Auezov South Kazakhstan University have contributed significantly to this Special Issue. The purpose of the current editorial article is to briefly summarize the articles included in the SI.

One of the most cost-effective approaches to obtaining sustainably built construction products is to research various types of recycling waste in construction and building materials. Klyuev et al. [1] proposed a method of obtaining mineral fiber fillers for dry building mixtures via the processing of waste from producing technogenic fibrous materials. These advanced materials are the continuation of the authors’ previous works [2,3,4,5,6] and represent exciting and essential results. Zhangabay et al. [7] presented the findings of experimental studies on the operational characteristics of prestressed shells constructed from recycled materials. These results summarize the findings of these Kazakh interuniversity researchers, published both in this Special Issue (to be discussed below) and in other publications [8,9,10,11,12,13]. Konovalova et al. [14] supported the use of fly ash in the composition of soil concrete, which is employed in the construction of structural layers of road pavements, foundations of buildings and structures, and sites for various uses. Pyagai et al. [15] investigated the effect of residual sulfuric and phosphoric acids on the process of converting significant amounts of phosphogypsum waste into calcium carbonate.

Aruralasi et al. [16] investigated the effects of admixtures on energy usage during the mixing of ready-mixed concrete. These findings are consistent with previous classic findings acquired by independent authors [17]. Volokitina et al. [18] examined the hardness of bimetallic wires made from secondary materials used in power line construction. Lisowska et al. [19] verified the hypothesis by assuming that combining waste sulfur pulp and its mixtures with organic materials enables simultaneous soil enrichment with readily available sulfur and organic matter. In [20], the study aims to predict the compressive strength of LFC by using a support vector machine as an individual learner along with bagging, boosting, and random forest as a modified ensemble learner. Khan et al. [21] evaluated the effect of polyethylene terephthalate alongside two supplementary cementitious materials—i.e., fly ash and silica fume—on the 28-day compressive strength of cementitious grouts for a semi-flexible pavement surface. Kasiman et al. [22] presented the mixed finite element formulation for examining the biomagnetic fluid dynamics as governed by the Navier–Stokes equation, coupled with energy and magnetic expressions. Yip et al. [23] researched a low-rise, three-story, reinforced concrete (RC) structure. This study aims to identify the dynamic response of the scaled RC structure with and without attached dampers, and it performs structural reliability of the tested model under the excitation of Peak Ground Acceleration of 0.1 g to 1.0 g with a unidirectional shaking table. Asghar et al. [24] presented a novel approach of artificial intelligence-based gene expression programming for predicting the lateral load-carrying capacity of RC rectangular columns when subjected to earthquake loading. The Kazakh team, led by Volokitina and Kolesnikov [25,26], presented studies on the possibility of utilizing technogenic waste from the metallurgical industry by the method of complex processing to reduce the anthropogenic load on the environment of the region, with the example given of a zinc silicate–magnetite–carbon system.

Ullah et al. [27] emphasized the mechanical properties of bituminous concrete mix prepared with crumb rubber and waste sugarcane bagasse ash. Loganina et al. [28] modified lime mortars with silicic acid sol to obtain more durable crystalline materials for construction purposes. Hakro et al. [29] studied the effect of marble dust, rice-husk ash, and saw dust on expansive shale’s compaction characteristics and permeability properties. These results are an excellent summary of many years of research by these authors [30,31,32,33,34]. Khanzada et al. [35] investigated the preplaced aggregate method of concrete utilizing silica fume and polypropylene fibers. Chin et al. [36] investigated the possibility of incorporating agro-industrial wastes into the brick mixture by examining their properties using several standardized tests. Smirnova et al. [37] compared the fracture features, strength, and deformation properties of pseudo-strain-hardening composites based on alkali-activated slag and Portland cement matrices with polypropylene microfiber. These results are an excellent summary of many years of research by these authors [38,39,40,41]. Alhokabi et al. [42] used the replacement portion of the soil with stabilizing agents such as palm oil fuel ash and gypsum. A team led by Niemiec [43,44] evaluated the efficiency of the conversion of biomass contained in the whitewater fraction of municipal waste and sewage sludge using methanogenesis. Ahmad et al. [45] used waste glass as a filler material that filled the voids between recycled concrete aggregate to offset its negative impact on concrete performance. Kolesnikov et al. [46] presented studies on the processing of enrichment tailings as a component of a raw mixture to obtain cement clinker with a simultaneous distillation of zinc. Campanhao et al. [47] developed recycled PET sand for cementitious mortar, whereas Chakrawarthi et al. [48] studied the impact resistance of polypropylene fiber-reinforced alkali-activated copper slag concrete. The authors of [49,50] demonstrated the application of a machine learning-based algorithm approach named “Multi Expression Programming” (MEP) to forecast the compressive strength of carbon fiber-reinforced polymer (CFRP)-confined concrete. Barabanshchikov et al. [51] studied the effect of a microfibrillated cellulose additive on strength, elastic modulus, heat release, and shrinkage of mortar and concrete. Petropavlovskii et al. [52] used gypsum-containing waste from a faience factory in the form of waste molds for casting dishes, souvenirs, and plumbing fixtures. Szelag et al. [53] presented the effect of brick powder (BP) on cement mortars’ physico-chemical and mechanical properties. Raza et al. [54] evaluated the moisture damage potential in hot mix asphalt using polymeric aggregate treatment. Alani et al. [55] investigated the demolition waste potential of completely cement-free binders. Al Salaheen et al. [56] researched on modelling and optimization for mortar compressive strength, incorporating heat-treated fly oil shale ash as an effective supplementary cementitious material using response surface methodology. Kolesnikova et al. [57] developed a thermodynamic simulation of environmental and population protection by utilization of technogenic enrichment tailings. All the research articles listed above are based on previously obtained experimental data from other publications [5,58,59,60,61,62,63,64,65].

A good embellishment of the Special Issue is several review articles. Ahmad et al. [66] presented a summary of research progress on coconut fiber (natural fibers)-reinforced concrete, whereas Shaikhiev et al. [67] provided a comprehensive review of the literature data on the use of apricot (Prunus armeniaca) biomass components as a sorption material for the treatment of wastewater and environmental water from various pollutants. Gul Zaman et al. [68] reviewed produced water treatment with conventional adsorbents and metal–organic frameworks as an alternative. Makul et al. [69] highlighted the overview of biomass energy in South East Asia as a dynamically developing region to obtain economic and environmental benefits from the existing biomass sources in the world. Finally, Marvila et al. [70] overviewed recycled aggregate as a viable solution for sustainable concrete production. The above-mentioned discussion is a brief overview of the published articles in the SI. In contrast, the editorial members and GE are organizing another SI for future new and unpublished work.
